# Prevalence of hepatitis C virus infection among HIV-infected people in Casablanca

**DOI:** 10.1186/1471-2334-14-S2-P20

**Published:** 2014-05-23

**Authors:** L Badaoui, G Dabo, R Bensghir, M Sodqi, L Marih, A Oulad Lahsen, A Chakib, K El Filali Marhoum

**Affiliations:** 1 University Hospital of Ibn rochd, Casablanca, Morocco

## Introduction

HIV/HCV coinfection may have important implications for therapeutic and evolving plans. The Knowledge of its epidemiological, clinical features may help to anticipate needs and improve care. The aim of the Study was to determine the epidemic profile of patients co- infected by hepatitis C virus and HIV.

## Materials and methods

A retrospective study was conducted in the infectious department diseases between 2003 and 201 3, were included all patients treated for HIV and HCV with positive serology (ELISA positive). Data were collected on computer files (Nadis) and analyzed on Epi Info.

## Results

We collected 147 patients with HIV/HCV coinfection (4% of the total workforce). The average age was 40 ± 10 years, males predominated (4H/1F). Intravenous drug use was found in 29.25 % of patients. At the moment of diagnosis of the coinfection, 64 patients (43.5%) were in category C (CDC) . Antiretroviral therapy was prescribed for 131 patients (89%). The viral RNA research by RT-PCR and the genotyping were performed in 39 patients (27 %). The RNA HCV PCR was positive in 28 cases (19 %). The most frequent viral genotypes were 1a and 1b with 14 cases (50%). The IL28B performed in 8 patients had type CC (2 cases), C/T (3 cases), TT (1 case). Liver function tests were normal in 116 cases; liver cytolysis was found in 31 cases. The liver ultrasound was normal (80 cases) or showed lesions of chronic liver disease (30 cases) or ascites (10 cases). Ten patients undergoing treatment with pegylated interferon and ribavirin. Two patients who completed treatment were non-responders. 79 patients (54%) are being used and 27 cases died (18%).

## Conclusion

The HIV/HCV co-morbidity is a challenge in support of PLHIV. Currently, free access to treatment and explorations of hepatitis C in Morocco as part of a health program (RAMED) is an important step whose impact needs to be evaluated.

